# Inhibition of IL‐17 Alleviates Blood–Brain Barrier Disruption Following Diffuse Axonal Injury Accompanied by Hyperglycemia Through the NF‐κB Pathway

**DOI:** 10.1155/mi/6634285

**Published:** 2026-05-28

**Authors:** Zhiguo Xing, Xing Wei, Ming Zhang, Tingqin Huang, Yonglin Zhao, Jinning Song

**Affiliations:** ^1^ Department of Neurosurgery, The First Affiliated Hospital of Xi’an Jiaotong University, Xi’an, 710061, China, xjtu.edu.cn; ^2^ Department of Neurosurgery, Weinan Central Hospital, Weinan, 714000, China; ^3^ Department of Gynaecology and Obstetrics, The Second Affiliated Hospital of Xi’an Jiaotong University, Xi’an, 710004, China, xjtu.edu.cn; ^4^ Department of Neurosurgery, The Second Affiliated Hospital of Xi’an Jiaotong University, Xi’an, 710004, China, xjtu.edu.cn; ^5^ Department of Comprehensive Breast Care Center, The Second Affiliated Hospital of Xi’an Jiaotong University, Xi’an, 710004, China, xjtu.edu.cn

**Keywords:** blood–brain barrier, diffuse axonal injury, hyperglycemia, interleukin-17A, nuclear factor kappa B

## Abstract

**Objective:**

To clarify the effects and mechanism of interleukin‐17A (IL‐17) in hyperglycemia (HG)‐induced blood–brain barrier (BBB) breakdown following diffuse axonal injury (DAI).

**Methods:**

Differentially expressed proteins (DEPs) were identified by proteomic analysis via 4D‐SmartDIA between control and high‐glucose groups of BBB model established by bEnd.3 cells in vitro. A rat model of DAI was built using an instantaneous rotational damage device, and HG was mimicked by intraperitoneal (i.p.) injection of 50% glucose. The localization and expression of the IL‐17 receptor (IL‐17R) were tested by double‐label immunofluorescence and western blotting. IL‐17 levels in brain tissue and serum, as well as the concentrations of inflammatory factors, were examined. Axonal injury morphology was evaluated via transmission electron microscopy (TEM) and immunohistochemical detection of β‐amyloid precursor protein (β‐APP) and neurofilament light chain and heavy chain (NF‐L, NF‐H). Glial response and apoptosis were also assessed. The detection of BBB permeability was via levels of Evans blue (EB) leakage and tight junction protein. The IL‐17 pathway was inhibited using suberoylanilide hydroxamic acid (SAHA), and western blotting was used to detect the phosphorylation (p‐p65/t‐p65 ratio) of the nuclear factor‐κB (NF‐κB) pathway. Oxidative stress levels were assessed via colorimetric assays.

**Results:**

Proteomic analysis revealed 444 upregulated and 159 downregulated proteins in high‐glucose‐stimulated bEnd.3 cells compared with those in normal cells, with the IL‐17 signaling being one of the most significantly enriched pathways according to the Kyoto Encyclopedia of Genes and Genomes (KEGG) analysis. IL‐17 and IL‐17R expression was elevated in brain tissue and serum following DAI, and HG further enhanced this upregulation; IL‐17R was predominantly localized to vascular endothelial cells. HG aggravated axonal injury, increased cortical apoptosis and glial response, promoted BBB disruption, elevated proinflammatory factor levels, and increased oxidative stress after DAI. The inhibition of IL‐17 reversed all the damage and protected BBB integrity by maintaining tight junctions and reducing the levels of proinflammatory factors and oxidative stress in vivo. Mechanistically, HG increased NF‐κB p65 phosphorylation after DAI, whereas SAHA treatment significantly suppressed this phosphorylation.

**Conclusion:**

IL‐17 mediates HG‐induced axonal injury following DAI by destroying BBB integrity via NF‐κB‐dependent inflammation and oxidative stress, signifying IL‐17 as a target for mitigating neurovascular damage in hyperglycemic DAI.

## 1. Introduction

Diffuse axonal injury (DAI), often resulting from rotational or acceleration–deceleration forces during trauma, is particularly devastating due to its complex pathophysiology and poor prognosis [1]. DAI is often accompanied by many complications, and one notable consequence is stress‐induced hyperglycemia (SHG). When DAI occurs, the body’s stress system is rapidly activated. Corticotropin‐releasing hormone and adrenocorticotropic hormone were secreted, leading to release of cortisol [2, 3]. This process, along with the suppression of insulin‐mediated glucose uptake by peripheral tissues, contributes to the elevation of blood glucose levels.

HG can exacerbate secondary brain injury after DAI [4]. SHG is closely linked to poor prognosis in traumatic brain injury (TBI) [5]. In clinical practice, the prognosis of DAI was not improved by intensive glycemic control [6]. High blood glucose levels after TBI fuel oxidative stress and exacerbate the inflammatory response. These processes damage neurons and accelerate cell death. Additionally, HG‐induced excitotoxicity, due to increased glutamate release, causes calcium overload in neurons, leading to irreversible damage [7]. All these factors contribute to more severe neurological deficits, longer hospital stays, and higher mortality rates. HG can disrupt the integrality of blood–brain barrier (BBB), resulting in secondary damage [8]. Disruption of the BBB allows the entry of inflammatory mediators and toxins into the brain parenchyma, aggravating the inflammatory response and tissue damage associated with TBI [9]. However, the mechanism by which HG damages the BBB remains unclear.

Interleukin‐17A (IL‐17), which is secreted by activated T helper 17 cells, is now recognized as a key proinflammatory cytokine in chronic immune‐mediated inflammatory diseases [10]. The levels of IL‐17 rapidly increase in injured brain tissue and serum, and inhibiting release of IL‐17 can improve the neurological function of rats [11]. Conversely, the levels of IL‐17 were distinctly reduced in the injured cortex after TBI. Moreover, neurological functioning was unchanged in IL‐17 knockout mice compared to normal mice [12]. The effects and mechanism of IL‐17 in secondary injury after DAI need to be further clarified.

In this research, we evaluated the distribution of the IL‐17 receptor (IL‐17R). Additionally, we investigated the impacts of an IL‐17 inhibitor on axonal injury following DAI complicated by HG. The influence of the IL‐17 inhibitor on the integrality of the BBB was also estimated. Our hypothesis was that HG subsequent to DAI triggered an increase in IL‐17 expression. The elevated IL‐17 further induced axonal injury, inflammation, and BBB compromise. Moreover, whether the harmful effects of IL‐17 are in connection with the nuclear factor‐kappa B (NF‐κB) pathway needs to be explored.

## 2. Materials and Methods

### 2.1. Cell Culture and Treatment

A bEnd.3 is an immortalized cerebral microvascular endothelial cell line with typical BBB phenotypic characteristics. bEnd.3 cells were cultured as monolayers in DMEM high‐glucose medium (with a glucose concentration of 25 mmol/L) at 37°C in an incubator with 5% CO_2_. When cells were 80% confluent, they were digested and seeded into coverslips in 24‐well plates, and the in vitro BBB model was successfully constructed. The cells were then randomized into high‐glucose group and control group. Cells in the high‐glucose group were put into a medium containing 200 mmol/L glucose, while cells in the control group were incubated under 25 mmol/L glucose at the same osmotic pressure for 24 h.

### 2.2. Measurement of Transendothelial Electrical Resistance (TEER) and Assessment of Horseradish Peroxidase (HRP) Permeability

When cell density was 2 × 10^5^ cells/mL, the cells were inoculated into the transwell plate. After cells formed a tight monolayer after 3–5 days, Millicell‐ERS instrument was used to examine TEER. The final TEER values were measured in μΩ/cm^2^ [13]. 2 μg/mL HRP was put into the upper chambers of transwell plates following by cell proliferation at 37°C. The medium in the lower chambers was collected and mixed with TMB substrate. The HRP flux was subsequently quantified and presented in units of nanograms per milliliter after terminating reaction by sulfuric acid [14].

### 2.3. Proteomics

The cells were lysed and sonicated. Equal amounts of protein were loaded after measuring the protein concentration. The proteins were reduced with dithiothreitol to break disulfide bonds and alkylated with iodoacetamide. Trypsin was then added at 37°C overnight, generating a mixture of peptides. The digested peptides were separated, eluted using a solvent gradient, and then introduced into the mass spectrometer. Precursor ion windows were then selected for MS/MS acquisition after a survey scan. Trapped ion mobility spectrometry was utilized to provide an additional fourth dimension for enhanced separation of the peptides. The data‐independent acquisition (DIA)‐NN search engine was used to process data analysis. Tandem mass spectra were searched, while several fixed modifications were included, such as excision on N‐term Met and carbamidomethyl on Cys. False discovery rate was adapted to <1%. The Kyoto Encyclopedia of Genes and Genomes (KEGG) pathway database was utilized to annotate protein pathways. BLAST comparison was used to identify proteins based on the comparison result with the highest score [15, 16]. On the basis of quantitative results, the fold change (FC) and the *p* value from the *t* test between the two groups were calculated to screen all differential proteins. The *p* value threshold was set as 0.05. A FC in differential expression that exceeded 1.5‐fold was considered statistically evident upregulation; conversely, a FC less than 1/1.5‐fold was considered downregulation.

### 2.4. Animal Grouping and Experimental Procedures

Sprague–Dawley (SD) male rats were from the Experimental Animal Center of Xi’an Jiaotong University. The weight and age of the rats were specified as 250–300 g and 8–10 weeks old. Prior to the study, the rats were acclimated under standard conditions, with a 12‐h photoperiod (light/dark) with the temperature sustained at 22 ± 0.5°C. 132 SD rats were randomized (via a random number table) into five groups. The following groups each consisted of 30 rats: control group, DAI 1 d group, DAI 1 d + HG group, and DAI 1 d + HG + suberoylanilide hydroxamic acid (SAHA, an IL‐23/IL‐17 axis inhibitor) group. In addition, there were 12 rats in DAI 3 d + HG group. HG was induced by intraperitoneal (i.p.) injection of 50% glucose (6.0 mL/kg) at 0 and 12 h postinjury. Tail vein blood samples were collected at 0, 12, and 24 h to monitor glucose levels, ensuring that concentrations remained >16.8 mmol/L within 24 h. At 4 h after DAI induction, the rats received i.p. injection of SAHA (Sigma‒Aldrich, SML0061, 50 mg/kg) or vehicle control (10% DMSO) on the basis of established protocols [17, 18].

### 2.5. Animal Model of DAI

DAI was induced by the lateral head rotation device according to previously established protocol [19]. Briefly, after successful anesthesia, rat head was horizontally fixed to the device using ear pins and a front teeth hole, with the body tilted at a 30° angle relative to the operating table. Trigger activation induced a 90° head rotation, subjecting the rats to abrupt acceleration–deceleration forces. Rats were only anesthetized without being subjected to injury in control group. In the DAI groups, the rats were allowed to acclimate for 30 min, followed by consciousness recovery. Vital signs were monitored during anesthetization to prevent asphyxiation. Postinjury, all injured rats presented a reduced stimulus response, unstable gait, and decreased activity/food intake for 12 h. Two rats that died postinjury were replaced with new rats.

### 2.6. Western Blot Analysis

After lysis, cortical brain protein samples were fractionated via SDS‐PAGE and subsequently electrophoretically transferred onto a PVDF membrane. The membranes were blocked and underwent incubation at 4°C for 12 h with the primary antibodies. Details are as follows: rabbit monoclonal IL‐17R (1:1000, Cell Signaling Technology (CST), Danvers, MA, USA), goat polyclonal ZO‐1 (1:1000, Abcam, Cambridge, UK), rabbit monoclonal claudin‐5 (1:1000, Abcam), rabbit monoclonal β‐actin (1:1000, CST), rabbit monoclonal occludin‐1 (1:500, Abcam), rabbit monoclonal NF‐κB p65 (1:1000, CST), and rabbit monoclonal phospho‐NF‐κB p65 (Ser536, 1:500, CST). After washing, the membranes were exposed to species‐specific secondary antibodies at 37°C for 1 h. The protein bands were visualized by the ChemiDoc MP System, and the densitometric values were normalized to those of the internal β‐actin controls [20].

### 2.7. Immunohistochemical Staining Protocols

Rat brains were dehydrated, paraffin‐embedded, and sectioned (5 µm). Antigen retrieval was implemented subsequent to deparaffinization and rehydration, prior to blocking endogenous peroxidase activity, followed by incubation with primary antibodies for 12 h. The specific antibody information is as follows: glial fibrillary acidic protein (GFAP, 1:500, CST), ionized calcium‐binding adapter molecule‐1 (Iba‐1, 1:400, Wako, Tokyo, Japan), β‐amyloid precursor protein (β‐APP, 1:400, Abcam), neurofilament light chain (NF‐L, 1:200, CST), and neurofilament heavy chain (NF‐H, 1:200, CST). After rinsing, the sections were treated by HRP‐conjugated secondary antibodies, and subsequently stained with 3,3^′^‐diaminobenzidine. The section was statistically analyzed in five random visual fields. Immunohistochemical scoring (IHS) was calculated as the product of the cell quantity score and the staining intensity score using Image‐Pro Plus 6.0 software. The quantity scores were defined as follows: zero, no staining; one, 1%–10% immunopositive cells; two, 11%–50% immunopositive cells; three, 51%–80% immunopositive cells; and four, 81%–100% immunopositive cells. The intensity scores were as follows: zero, negative; one, weak staining; two, moderate staining; and three, strong staining. The theoretical score range of IHS is zero to twelve (0–12). All sections were evaluated by an experienced pathologist blinded to the experimental groups [14].

### 2.8. Transmission Electron Microscopy (TEM)

Samples for TEM were prepared following established protocols [21]. Cortical tissues of interest were dissected into 1 mm^3^ blocks, postfixed at 4°C, and processed as follows: (1) Fixation and washing: after fixation in 1% osmium tetroxide for 2 h, the samples were then washed with PBS for 10 min. (2) Dehydration and embedding: dehydration was achieved through an ethanol gradient series, Epon 812 resin was used to embed the samples, and samples were cut into semithin sections (2 μm). (3) Section staining and imaging: Semithin sections were treated with methylene blue and then trimmed for ultrathin sectioning (50 nm) using an ultramicrotome. After counterstained with 2% uranyl acetate and followed by 3% lead citrate, the sections were imaged via TEM.

### 2.9. Immunofluorescence Staining

The brain tissue sections were processed using the same deparaffinization and rehydration steps as those described for immunohistochemistry. The staining protocol was mainly the same as immunohistochemistry. The primary antibodies were rabbit polyclonal IL‐7R (1:400, CST), mouse monoclonal NeuN (1:400, Millipore), mouse monoclonal GFAP (1:400, CST), mouse monoclonal CD34 (1:400, Invitrogen, Carlsbad, CA, USA), and goat polyclonal Iba‐1 (1:200, Abcam). Subsequently, the sections were treated with Alexa 488/568‐conjugated secondary antibodies (1:400, Invitrogen) at 37°C for 1 h, and cell nuclei were treated with 4^′^, 6‐diamidino‐2‐phenylindole (DAPI) for 10 min. Sections were then visualized under a fluorescence microscope [13]. To remove the observer bias, acquisition blinding, analysis blinding, and unified standardization were adopted throughout the image acquisition and colocalization determination.

### 2.10. Hematoxylin and Eosin (H&E) Staining

Rat brain samples were dehydrated, paraffin‐embedded, and sectioned the same as immunohistochemistry. Sections were first treated by H&E, and then, sections undergone dehydration, hyalinization, and mounting. Three sections per animal were observed and analyzed.

### 2.11. Terminal Deoxynucleotidyl Transferase‐Mediated Digoxigenin‐dUTP‐Biotin Nick‐End Labeling (TUNEL) Assay

The DeadEnd Fluorometric TUNEL System (Promega, Madison, WI, USA) was performed to detect cell apoptosis. Brain tissue sections were prepared as described for immunohistochemistry. 20 μg/mL proteinase K was applied to incubate the sections for 10 min. DAPI was adopted to stain nuclei, and sections were visualized under a fluorescence microscope. The apoptotic rate was defined as the ratio of TUNEL‐positive cells to the total number of DAPI‐stained nuclei [13].

### 2.12. Enzyme‐Linked Immunosorbent Assay (ELISA)

The total protein concentration was quantified after homogenization and centrifugation. Inflammatory cytokines (tumor necrosis factor‐α [TNF‐α], interleukin‐1β [IL‐1β], and interleukin‐6 [IL‐6]) in cortical lysates were measured following the manufacturer’s protocol. The samples were assayed in duplicate, and the cytokine levels (pg protein) were normalized to the total protein concentration (mg) [21].

### 2.13. BBB Permeability Assay

2% (w/v) Evans blue (EB) was dissolved and administered intravenously (4 mL/kg) 1 h prior to tissue collection. Brain tissues were homogenized and precipitated with 50% trichloroacetic acid for 12 h. The supernatant was acquired subsequent to centrifugation. Absorbance was quantified at 610 nm by a spectrophotometer. EB extravasation was calculated as micrograms of EB per gram of brain tissue.

### 2.14. Wet–Dry Method to Test Brain Edema

The protocol of standard wet–dry method involved the following steps: (1) The brain was weighed and the wet weight was obtained. (2) The brain was dried at 105°C for 3 days, after which the dry weight was measured. The BWC value was quantified via the following formula [22]:

BWC=wet weight − dry weight/wet weight×100%.

### 2.15. Detection of Oxidative Stress

After homogenized and centrifuged, the protein concentration was quantified. Tissue lysates were then analyzed to measure the activities of oxidative stress biomarkers‐malondialdehyde (MDA), superoxide dismutase (SOD), glutathione peroxidase (GPx), and catalase (CAT), following the manufacturer’s protocols. Standard curves were performed to calculate the concentrations [14].

### 2.16. Statistical Analysis

SPSS 18.0 was used to perform statistical analyses. The data were shown as the means ± SDs. Statistical significance was defined as *p* < 0.05. Statistical analysis of biochemical data was done using one‐way ANOVA with Tukey’s post hoc test. All significant differences are visualized in bar graphs.

## 3. Results

### 3.1. High Glucose Impaired the Integrality of the BBB In Vitro

**Figure 1:**
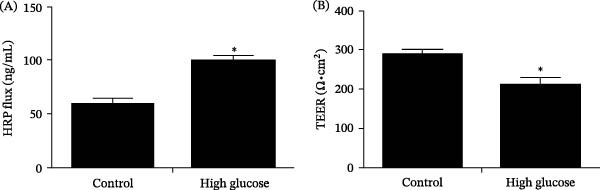
In vitro, high‐glucose treatment impaired BBB permeability, as verified by HRP flux (A) and TEER measurements (B).  ^∗^ *p* < 0.05, relative to control group.

Compared with the control group, the high‐glucose group showed a decrease in TEER and an increase in HRP flux. The data imply that high glucose can compromise the permeability of the BBB (Figure 1).

### 3.2. Proteomic Analysis Using 4D‐SmartDIA to Identify Differentially Expressed Proteins (DEPs)

**Figure 2:**
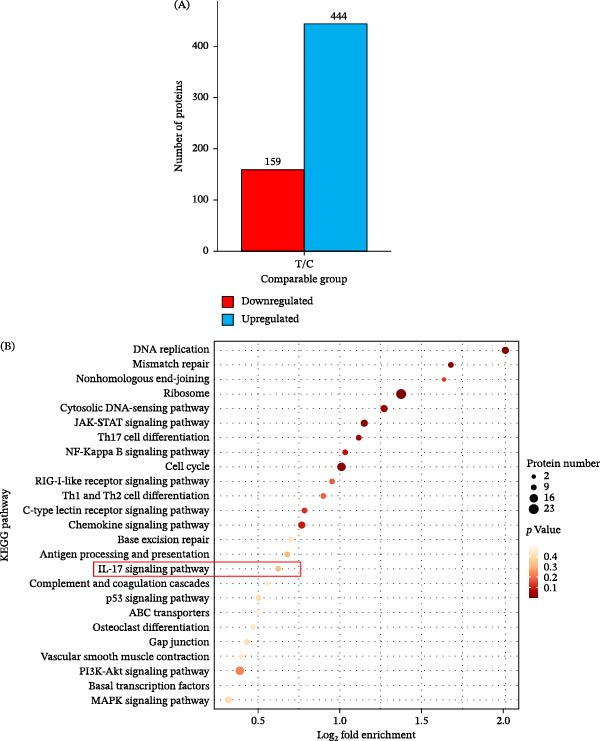
DEPs were acquired by proteomic analysis between the control and high‐glucose groups. (A) Bar plot showing DEPs between the two groups via 4D‐SmartDIA (*n* = 3). (B) Statistical analysis of pathway enrichment for DEPs in pairwise comparisons. *X*‐axis: log_2_‐transformed fold enrichment of functional enrichment; *Y*‐axis: KEGG functional descriptions.

4D‐SmartDIA was used to perform proteomic analysis between control group and high‐glucose group. A total of 603 DEPs were identified. Amidst all the DEPs, 444 were elevated, and 159 were downregulated (Figure 2A). A scatter plot of the top 25 pathways was acquired and annotated by KEGG pathway enrichment. The IL‐17 signaling pathway was one of the most significantly enriched pathways (Figure 2B).

### 3.3. HG Further Heightened the Level of IL‐17 and IL‐17R in Rats After DAI

**Figure 3:**
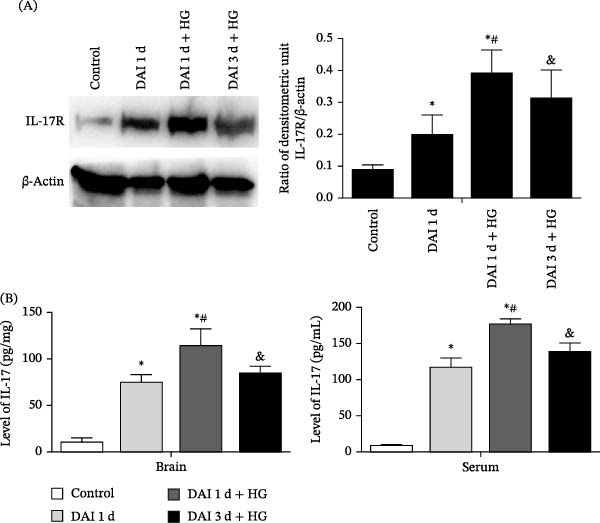
Dynamic expression of IL‐17R and IL‐17 in the brain and serum following DAI combined with hyperglycemia. (A) The dynamic expression of IL‐17R. (B) ELISA measurements of IL‐17 in cortex and serum.  ^∗^ *p* < 0.05, relative to control group; ^#^ *p* < 0.05, relative to DAI 1 d group; ^&^ *p* < 0.05 relative to DAI 1 d + HG group.

In comparison with the DAI 1 d group, HG exacerbated the increase in IL‐17R expression. In contrast, the expression of IL‐17R in DAI 3 d + HG group was lower compared to DAI 1 d + HG group. The ELISA results revealed that the levels of IL‐17 in both brain and serum were elevated in the DAI 1 d group compared to control group. The levels of IL‐17 in both brain and serum were higher after HG treatment than that in DAI 1 d group. Additionally, compared to DAI 1 d + HG group, the levels of IL‐17 were lower in DAI 3 d + HG group (Figure 3).

### 3.4. Localization of IL‐17R After DAI in Combination With HG in the Cortex of Rats

**Figure 4:**
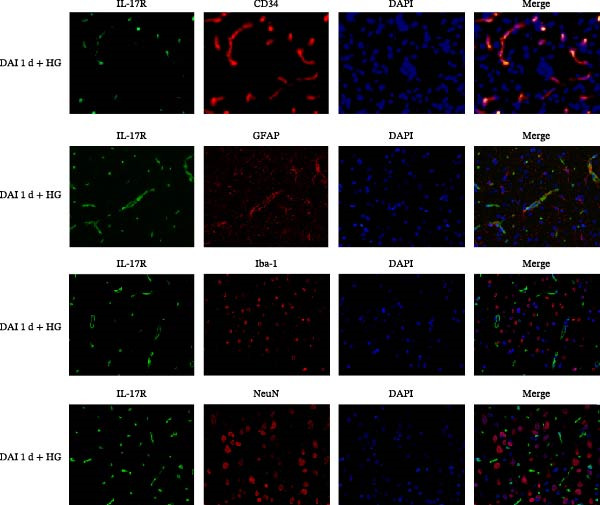
Localization of IL‐17R in various cell types following DAI combined with hyperglycemia. Cell‐specific markers: red (GFAP for astrocytes, NeuN for neurons, CD34 for vascular endothelial cells and Iba‐1 for microglia), green (IL‐17R), blue (nuclei). Scale bar = 50 μm.

To determine the localization of IL‐17R, double‐labeling experiments were carried out. The double‐labeling experiments involved Iba‐1 (indicator for microglia), GFAP (indicator for astrocytes), NeuN (indicator for neuronal nuclei), CD34 (indicator for vascular endothelial cells), and IL‐17R. Through double immunostaining, IL‐17R‐ and GFAP‐positive astrocytes, IL‐17R‐ and Iba‐1‐positive microglia, and IL‐17R‐ and NeuN‐positive neurons were scarcely detected. Surprisingly, in DAI 1 d + HG group, the colocalization of IL‐17R and CD34 by double‐label immunofluorescence was prominent. The results indicated that IL‐17R was expressed mainly in vessel endothelial cells after DAI in combination with HG (Figure 4).

### 3.5. Inhibition of IL‐17 Relieved Nerve Cell Damage After DAI in Combination With HG

**Figure 5:**
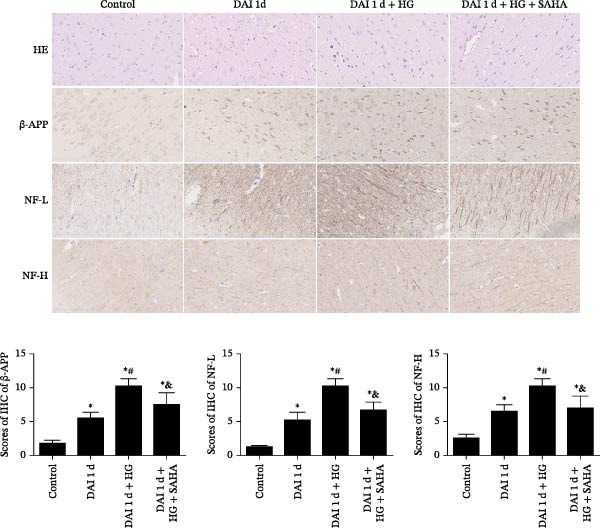
Role of IL‐17 on axonal injury following DAI combined with hyperglycemia (scale bar = 100 μm).  ^∗^ *p* < 0.05, relative to control group; ^#^ *p* < 0.05, relative to DAI 1 d group; ^&^ *p* < 0.05, relative to DAI 1 d + HG group.

In the H&E sections, the cell architecture was intact in normal rats. Neuronal pyknosis, torsion, and several abnormal pathological manifestations were observed after DAI. Compared to DAI 1 d group, all the pathological damage was more severe after management of HG. In contrast, compared to DAI 1 d + HG group, the inhibition of IL‐17 by SAHA alleviated the abnormal histopathological changes (Figure 5). The expression of axonal injury markers, including NF‐L, NF‐H, and β‐APP were scarcely detected in control group and elevated in DAI 1 d group. Notably, compared with DAI 1 d group, the HG treatment presented increased expression of axonal injury markers. Compared to DAI 1 d + HG group, the expression of aforementioned markers of axonal injury in the DAI 1 d + HG + SAHA group was significantly lower (Figure 5).

**Figure 6:**
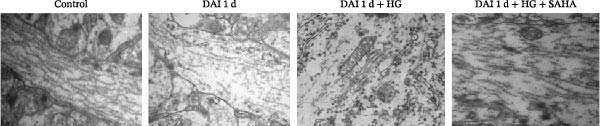
Role of IL‐17 in axonal ultrastructural alterations following DAI combined with hyperglycemia assessed by TEM (scale bar = 500 nm).

The TEM showed that the morphology of microtubules and mitochondria was continuous and undamaged in control group. In the DAI 1 d group, the microtubule structure was frayed, and the mitochondrial structure disappeared. Moreover, damage to microtubules and mitochondria was more pronounced after management of HG. In the DAI 1 d + HG + SAHA group, the inhibition of IL‐17 led to partial preservation of axonal continuity (Figure 6).

### 3.6. Impacts of HG and IL‐17 Inhibition on Glial Responses and Cell Apoptosis

**Figure 7:**
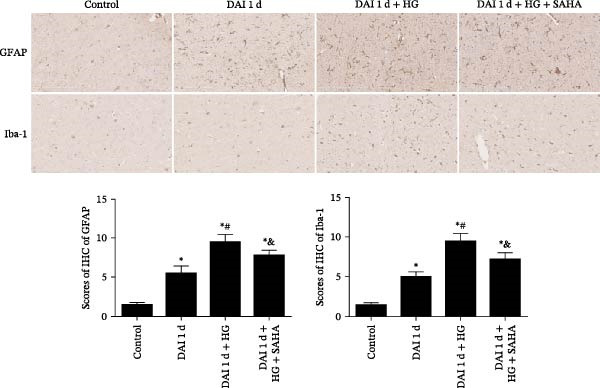
Effects of IL‐17 inhibitor on abundance of glial response (scale bar = 100 μm) following DAI combined with hyperglycemia.  ^∗^ *p* < 0.05, relative to control group; ^#^ *p* < 0.05, relative to DAI 1 d group; ^&^ *p* < 0.05, relative to DAI 1 d + HG group.

**Figure 8:**
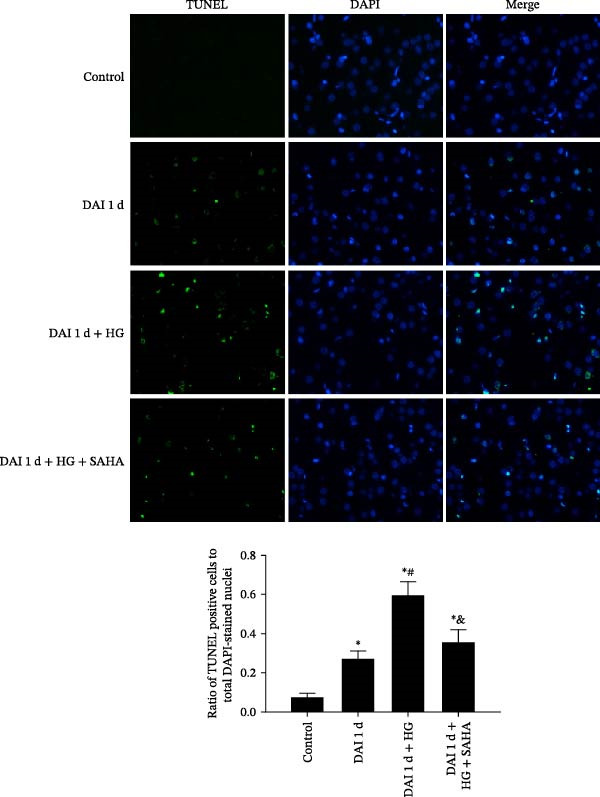
Hyperglycemia aggravated cell apoptosis and inhibition of IL‐17 could alleviate hyperglycemia‐induced cell apoptosis after DAI (40× magnification).  ^∗^ *p* < 0.05, relative to control group; ^#^ *p* < 0.05, relative to DAI 1 d group; ^&^ *p* < 0.05, relative to DAI 1 d + HG group.

In control group, the expression of glial response markers, including Iba‐1 and GFAP, was extremely low, and apoptotic cells were rarely detected in the cortex. Compared with control group, the expression levels of Iba‐1 and GFAP, as well as the ratio of TUNEL‐positive cells to total DAPI‐stained nuclei in the cortex, were significantly greater in the DAI 1 d group. After HG treatment, the expression of Iba‐1 and GFAP, and apoptosis rate were further increased. Notably, SAHA significantly decreased the abundance of glial response markers and apoptosis rate in the cortex at 1 d after DAI in hyperglycemic condition (Figures 7 and 8).

### 3.7. Role of IL‐17 in Damage of BBB Integrality After DAI Combined With HG

**Figure 9:**
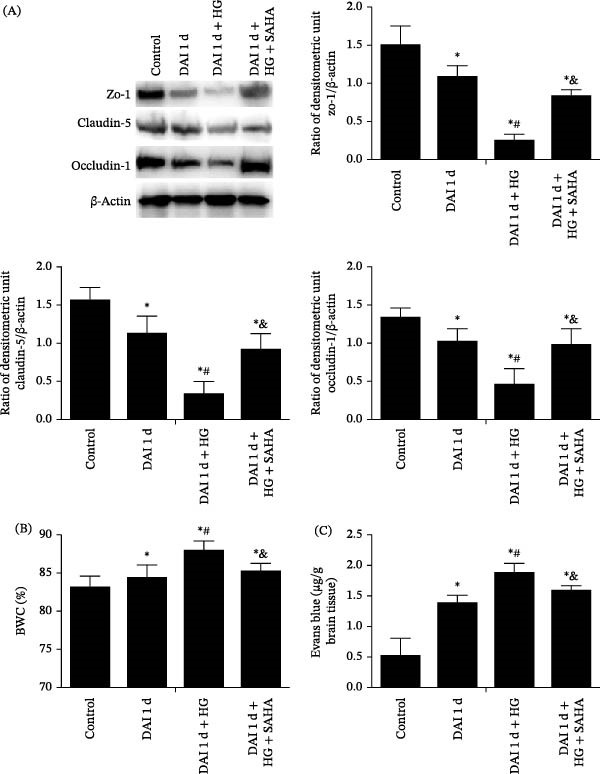
Role of IL‐17 in regulating BBB permeability following DAI combined with hyperglycemia. (A) The abundance of tight junction proteins was assessed. (B, C) BWC and Evans blue extravasation were measured to evaluate BBB permeability.  ^∗^ *p* < 0.05, relative to control group; ^#^ *p* < 0.05, relative to DAI 1 d group; ^&^ *p* < 0.05, relative to DAI 1 d + HG group.

The abundance of tight junction proteins, including claudin‐5, ZO‐1, and occludin‐1, was lower after DAI compared to control group. Compared to DAI 1 d group, HG exacerbated the reduction in tight junction proteins expression. Conversely, SAHA treatment upregulated the expression of tight junction proteins compared with that in the DAI 1 d + HG group. Compared with the control group, DAI led to brain edema and EB leakage. HG treatment further intensified brain edema and EB leakage compared to DAI 1 d group. However, SAHA treatment mitigated brain edema and EB diffusion (Figure 9).

### 3.8. Inhibition of IL‐17 Suppressed the Levels of Proinflammatory Factors and Oxidative Stress via the NF‐κB Pathway After DAI in Combination With HG

**Figure 10:**
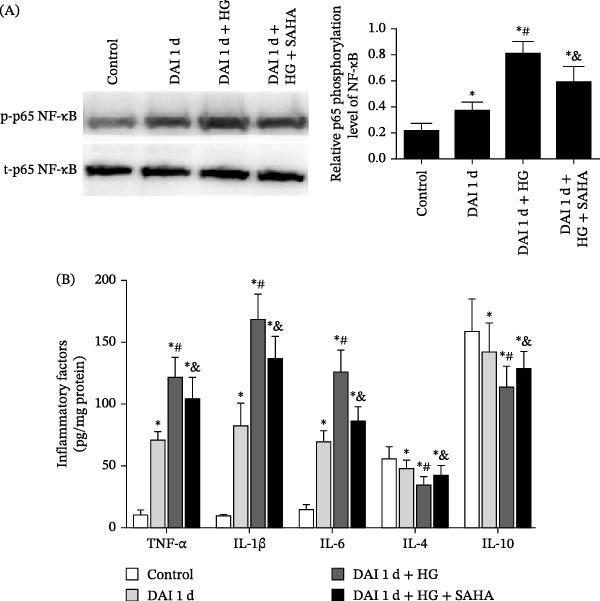
Inhibition of IL‐17 by SAHA reduced NF‐κB phosphorylation and the levels of proinflammatory factors and increased the levels of anti‐inflammatory factors in hyperglycemic rats at 1 day after DAI. (A) The phosphorylation level of NF‐κB was assessed. (B) Effects of hyperglycemia and the IL‐17 inhibitor on the abundance of TNF‐α, IL‐1β, IL‐6, IL‐4, and IL‐10 after DAI.  ^∗^ *p* < 0.05, relative to control group; ^#^ *p* < 0.05, relative to DAI 1 d group; ^&^ *p* < 0.05, relative to DAI 1 d + HG group.

**Figure 11:**
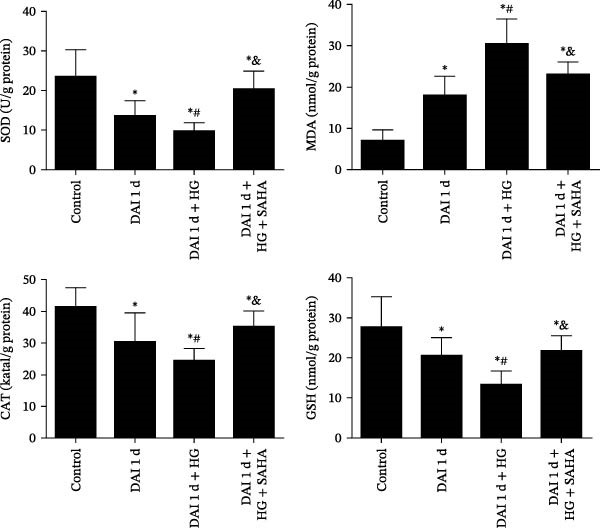
Role of IL‐17 in oxidative stress, as reflected by abundance of SOD, CAT, MDA, and GSH, in hyperglycemic rats at 1 day after DAI.  ^∗^ *p* < 0.05, relative to control group; ^#^ *p* < 0.05, relative to DAI 1 d group; ^&^ *p* < 0.05, relative to DAI 1 d + HG group.

The phosphorylation level of NF‐κB was evaluated by calculating the ratio of the expression of phosphorylated p65 NF‐κB (p‐p65 NF‐κB) to that of total p65 NF‐κB (t‐p65 NF‐κB). The phosphorylation level of NF‐κB was enhanced after DAI compared to control group. Compared to DAI 1 d group, the phosphorylation level of NF‐κB was further increased after HG treatment. Conversely, SAHA treatment led to a decrease in NF‐κB phosphorylation compared to DAI 1 d + HG group. Compared to control group, the levels of proinflammatory factors (TNF‐α, IL‐1β, and IL‐6) after DAI were elevated, whereas the levels of anti‐inflammatory factors (IL‐4 and IL‐10) were decreased. The DAI 1 d + HG group presented higher levels of proinflammatory factors and lower levels of anti‐inflammatory factors than DAI 1 d group. In contrast, SAHA treatment attenuated neuroinflammation by reducing pro‐inflammatory factors and restored anti‐inflammatory homeostasis in the cortex compared with those in the DAI 1 d + HG group (Figure 10). Compared to control group, the abundance of MDA was raised, whereas the abundance of SOD, GSH, and CAT reduced after DAI. HG treatment presented an even higher abundance of MDA and lower abundance of SOD, GSH, and CAT than DAI 1 d group. However, in the DAI 1 d + HG + SAHA group, SAHA effectively decreased the MDA level and increased the abundance of SOD, GSH, and CAT (Figure 11).

## 4. Discussion

HG is prevalent in patients after TBI and is correlated with aggravated all‐cause mortality. It induces endothelial cell damage through mechanisms such as polyol pathway activation and the accumulation of advanced glycation end products [23]. Additionally, HG exacerbates oxidative stress and inflammation, disrupting the endothelial barrier and increasing vascular permeability [8]. Previous studies have demonstrated that BBB disruption after DAI was aggravated by HG through upregulating the expression of inflammatory mediators. However, the underlying mechanisms remain incompletely understood [4, 19]. In the present study, proteomic analysis of bEnd.3 cells with or without high‐glucose stimulation revealed the IL‐17 signaling pathway as a key differentially expressed pathway. Consistent with these findings, elevated levels of IL‐17 and its receptor (IL‐17R) were observed in both brain tissue and serum. Functionally, inhibiting IL‐17 alleviated axonal injury, reduced glial activation, and decreased cortical apoptosis in hyperglycemic rats after DAI. Mechanistically, our findings suggest that HG may disrupt the BBB via the IL‐17/NF‐κB pathway, thereby promoting the release of inflammatory factors and increasing oxidative stress following DAI.

The role of IL‐17 in TBI, particularly its expression dynamics, remains controversial. One study reported that IL‐17 levels in the brain and serum increase from 6 h to 7 days postinjury, peaking at 3 days, with this trend paralleling the severity of secondary damage [11]. In contrast, in a TBI model induced by cortical impact, IL‐17 expression was significantly downregulated in the ipsilateral cortex and hippocampus by day 3 and remained low for up to 28 days [12]. Notably, the specific role of IL‐17 in secondary axonal injury after DAI has been poorly characterized. Our study clarified the expression patterns of IL‐17 in the context of DAI: we observed increased IL‐17 levels and IL‐17R expression in both the brain and serum post‐DAI, with IL‐17R predominantly localized to vascular endothelial cells. Moreover, HG further elevated IL‐17 levels, which gradually declined over time, confirming the selection of the DAI 1 d + HG group for subsequent experiments.

Axonal injury is characterized by the accumulation of axonal injury markers, which reflect impaired axonal transport [19]. In the present study, HG exacerbated pathological damage, increased apoptotic cell counts, and upregulated axonal injury marker expression after DAI, indicating that post‐DAI HG aggravated axonal injury and neurological dysfunction in rats. Conversely, inhibition of IL‐17 with SAHA alleviated neuronal damage, reduced apoptosis, and downregulated β‐APP and neurofilament expression in hyperglycemic DAI models, highlighting the neuroprotective effects of SAHA under these conditions. These results align with previous research showing that IL‐17 inhibition reduces IL‐17 levels, mitigates neuronal apoptosis, and improves neurological function after TBI [11]. However, conflicting evidence exists: no differences in neurological function were observed between IL‐17 knockout and wild‐type mice, and genetic deletion of IL‐17 failed to confer neuroprotection after TBI [12]. This discrepancy may reflect context‐dependent roles of IL‐17 in injury pathophysiology, potentially influenced by injury severity, timing, or comorbidities such as HG. Additionally, SAHA has been shown to protect against ischemic brain damage in hypertensive rats by improving neurological scores, reducing infarct volume and edema, suppressing microglial activation, and preserving BBB integrity, further supporting its potential as a neuroprotective agent in neurotrauma [18]. Collectively, these findings confirm that IL‐17 is activated in hyperglycemic DAI and contributes to the exacerbation of neuronal damage.

Upon binding to its receptor, IL‐17 recruits the cytoplasmic adaptor Act1, triggering NF‐κB activation. Activated NF‐κB then upregulates the transcription of proinflammatory and antimicrobial genes. Concurrently, the IL‐17 activates mitogen‐activated protein kinases and activator proteins, amplifying inflammatory cascades [17, 18]. In the present study, HG increased NF‐κB phosphorylation (assessed by the p‐p65/t‐p65 ratio) and elevated the levels of proinflammatory cytokines, whereas SAHA downregulated the generation of proinflammatory factors, accompanied by increased levels of anti‐inflammatory cytokines via NF‐κB pathway. These results align with previous reports that NF‐κB blockade mitigates experimental autoimmune encephalomyelitis by reducing antigen‐specific T‐cell responses and IL‐17 production and that Cordyceps polysaccharide attenuates ischemic brain injury by inhibiting NF‐κB and the IL‐23/IL‐17 axis, thereby decreasing the levels of proinflammatory cytokines [24, 25]. Together, these findings highlight the critical role of IL‐17/NF‐κB‐mediated inflammation in the pathophysiology of HG‐exacerbated DAI.

The BBB maintains brain homeostasis by restricting the entry of peripheral inflammatory factors while permitting nutrient transport. Inflammatory cytokines compromise BBB permeability, facilitating neuroinflammation, whereas BBB damage impairs the supply of protective factors (e.g., neurotrophins and antioxidant enzymes), exacerbating neuronal injury [26]. Our study demonstrated that DAI disrupts the BBB and that HG further exacerbates this damage. IL‐17 inhibition mitigated the damage of tight junction, thereby maintaining BBB integrity. These results support previous findings in the literature that IL‐17 blockade protects the BBB; for example, inhibiting Th17 differentiation via JAK1‐STAT6 signaling maintains BBB integrity and alleviates depression‐like behavior in fluoxetine‐resistant models, and in epilepsy, IL‐17R knockout suppresses Src kinase activation, whereas the Src inhibitor PP1 mitigates IL‐17A‐induced endothelial injury and BBB disruption [27, 28]. Collectively, these results establish that IL‐17‐driven inflammation is a key mediator of BBB breakdown in neurological injury.

SAHA, a zinc‐dependent class I/IIb histone deacetylase (HDAC) inhibitor, has been proved to enhance neuroplasticity and promote functional recovery after photothrombotic focal cortical stroke [29]. A previous study investigated the effect of SAHA on IL‐12p40‐related cytokine expression and reported that it suppresses IL‐23p19 gene transcription [30]. This inhibition may promote inflammatory/autoimmune diseases mediated by Th17 responses, suggesting that SAHA could ameliorate TBI by blocking the IL‐23/IL‐17 axis [11]. SAHA exerts neuroprotective effects through two pathways: (1) Against BBB disruption: The present study revealed that in hyperglycemic DAI, SAHA mitigates IL‐17‐driven inflammation and oxidative stress, preserving tight junction proteins. (2) Via oxidative stress control: In rat TBI models, SAHA inhibited HDAC1/3 and NADPH oxidase 4 (Nox4) in the injured cortex. Together, Nox4 suppression reduces local oxidative stress [31]. However, whether SAHA exerts additional neuroprotective effects through other pathways remains to be investigated.

In summary, our proteomic analysis identified the IL‐17 signaling pathway as a pivotal differentially expressed pathway in HG‐exacerbated DAI. Elevated levels of IL‐17 and its receptor link HG to IL‐17‐mediated inflammation after DAI. Inhibition of IL‐17 by SAHA mitigated axonal damage, suppressed glial activation, and reduced cortical apoptosis in a rat model of HG‐complicated DAI. Mechanistically, HG promotes BBB disruption via the IL‐17/NF‐κB signaling cascade, exacerbating the release of proinflammatory factors and oxidative stress after DAI. These findings identify IL‐17 as a novel target with therapeutic implications for preventing BBB breakdown and ameliorating hyperglycemic exacerbation in neurotrauma.

## Author Contributions

Zhiguo Xing and Xing Wei drafted the initial manuscript. Zhiguo Xing, Xing Wei, Ming Zhang, and Tingqin Huang conceptualized the study and developed its preliminary framework. Ming Zhang and Tingqin Huang were responsible for data collection and preparation. Yonglin Zhao and Jinning Song critically revised the manuscript and finalized the results.

## Funding

The National Natural Science Foundation of China (Grant 82001327) supported this research.

## Ethics Statement

This study was approved by the Biomedical Ethics Committee of the Health Science Center, Xi’an Jiaotong University (Approval Number XJTUAE2025‐1241). No human subjects or human tissues were involved in the study.

## Consent

All authors have consented to the publication of this work.

## Conflicts of Interest

he authors declare no conflicts of interest.

## Data Availability

The datasets generated and/or analyzed in the present study are available from the corresponding author upon reasonable request.
